# Association Between Serum Magnesium Levels and Metabolic Dysfunction-Associated Steatotic Liver Disease in a Nationally Representative Sample of Korean Adults, 2024

**DOI:** 10.3390/metabo16080583

**Published:** 2026-08-18

**Authors:** Seong-Uk Baek, Jin-Ha Yoon

**Affiliations:** 1 Graduate School, Yonsei University College of Medicine, Seoul 03722, Republic of Korea; 2 Institute for Innovation in Digital Healthcare, Yonsei University Health System, Seoul 03722, Republic of Korea; 3 The Institute for Occupational Health, Yonsei University College of Medicine, Seoul 03722, Republic of Korea; 4 Department of Preventive Medicine, Yonsei University College of Medicine, Seoul 03722, Republic of Korea

**Keywords:** metabolic dysfunction, metabolic syndrome, fatty liver disease, Mg, epidemiology, MASLD prevention

## Abstract

**Background/Objectives:** Magnesium (Mg) deficiency has been linked to various adverse health outcomes; however, its association with metabolic dysfunction-associated steatotic liver disease (MASLD) has not been fully elucidated. This study examined the association of serum Mg levels with MASLD in Korean adults. **Methods:** We analyzed a nationwide sample of 4952 adults in South Korea. Serum Mg levels (mg/dL) were measured. MASLD was defined as a hepatic steatosis index score of ≥36 together with the presence of cardiometabolic risk factor, including overweight/obesity, high fasting glucose, high blood pressure, high plasma triglycerides, or low plasma HDL cholesterol. The association between serum Mg levels and MASLD was examined using logistic regressions. Odds ratios (ORs) and 95% confidence intervals (CIs) were determined. **Results:** The mean (standard deviation [SD]) serum Mg level was 2.13 (0.15) mg/dL. The prevalence of MASLD was 25.7%. After adjusting for the sociodemographic variables, the OR (95% CI) for the association between a 1–SD increment in serum Mg levels and MASLD was 0.90 (0.84–0.96, *p* = 0.004). **Conclusions:** This nationally representative cross-sectional study found that serum Mg levels were inversely associated with the likelihood of MASLD among adults in South Korea.

## 1. Introduction

The public health burden of metabolic dysfunction-associated steatotic liver disease (MASLD) has substantially risen in recent years. Following the shift from non-alcoholic fatty liver disease (NAFLD) to MASLD, which emphasizes a positive definition based on metabolic abnormalities and adopts non-stigmatizing terminology [1], the prevention and management of MASLD have received increasing attention [2,3]. The prevalence of MASLD has gradually increased globally [4], with a similar upward trend observed in South Korea [5,6].

Magnesium (Mg) is a vital cation required for various physiological processes, primarily because of its role as a cofactor in numerous enzymatic reactions [7,8]. Previous studies have linked Mg deficiency to various adverse health outcomes, including cardiovascular disease onset and mortality risk [9,10]. However, owing to the increased consumption of processed foods and reduced intake of vegetables, fruits, and whole grains, suboptimal Mg intake has become widespread in both South Korea and global populations [11,12].

Previous studies have documented that various factors, including lifestyles, occupational exposure, and environmental exposure, can affect cardio-metabolic dysfunction and steatotic liver disease [13,14,15,16]. However, research examining the relationship between Mg levels and MASLD is scarce. A cross-sectional analysis of adults in the United States (US) reported that the Mg depletion score, calculated based on diuretic use, proton-pump inhibitor use, kidney function, and alcohol use, was positively associated with MASLD [17]. The Mg depletion score was also directly associated with mortality risk among individuals with MASLD [18]. Similarly, another study showed a direct association between the Mg depletion score, Mg intake, and MASLD [19]. Earlier studies reported that Mg intake was inversely related to the risk of hepatic steatosis or NAFLD in adults in the US [20,21,22]. A study of 226 healthy liver donors in Iran found that higher serum Mg levels were linked to a lower likelihood of NAFLD [23]. The mechanisms underlying the association between Mg status and MASLD have not yet been fully elucidated. Mg deficiency has been hypothesized to contribute to the pathogenesis of MASLD through multiple mechanisms, including insulin resistance, systemic inflammation, and metabolic dysfunction [24].

Despite existing evidence on the association of Mg status with MASLD, several important research gaps remain. First, most previous studies have assessed Mg status using dietary Mg intake or proxy measures, such as the Mg depletion score [17,18,19,20,21,22], whereas studies investigating the association of serum Mg levels with MASLD remain relatively scarce [23]. Second, existing evidence is largely limited to the US and is primarily based on datasets from the National Health and Nutrition Examination Survey (NHANES). To our knowledge, the association between serum Mg levels and MASLD has not yet been investigated in South Korea or, more broadly, in Asian populations. Therefore, we explored the relationship between serum magnesium levels and MASLD using a nationally representative sample of Korean adults.

## 2. Methods

### 2.1. Study Population

**Figure 1:**
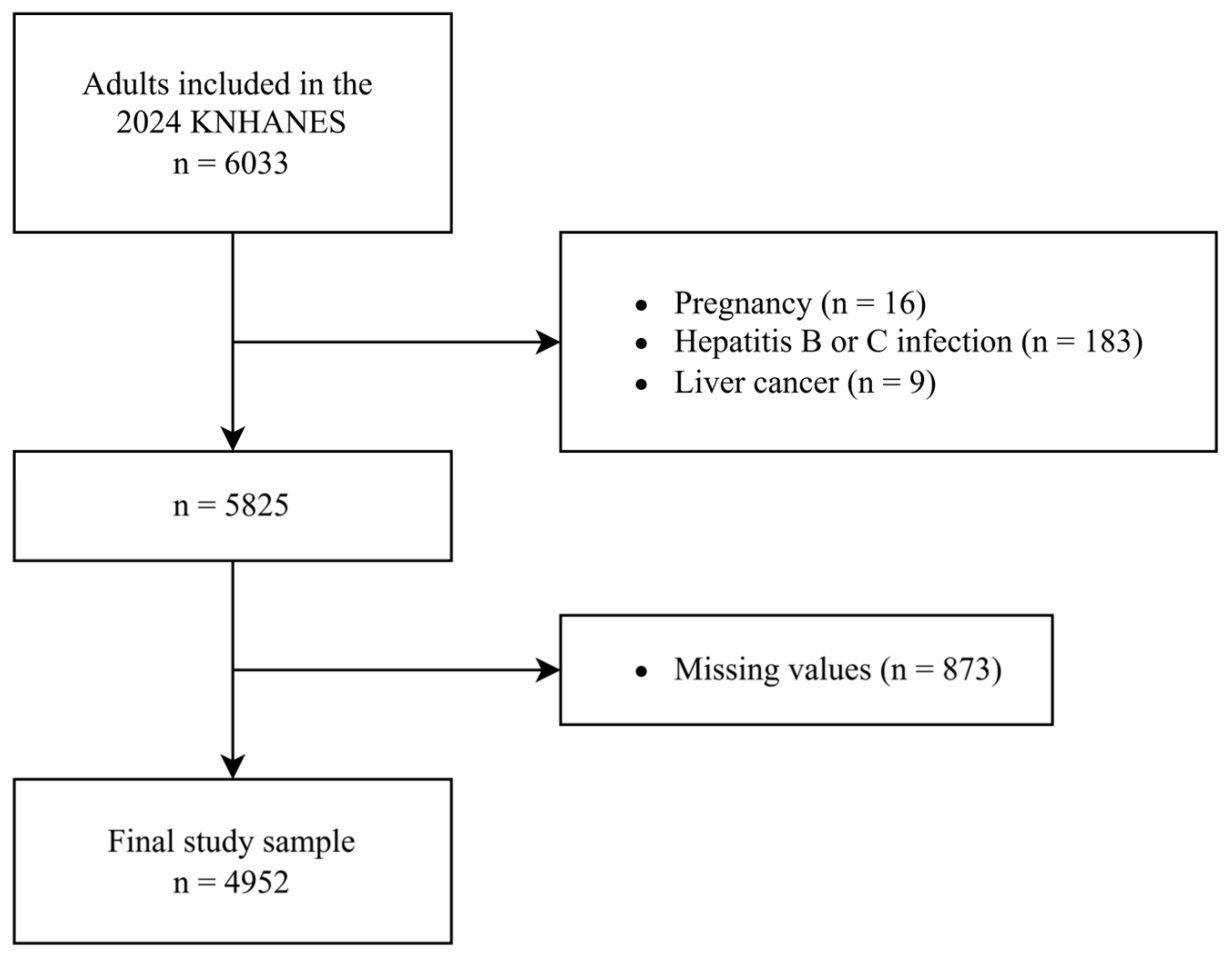
Study sample selection (Flowchart).

This study used data from the Korean NHANES (KNHANES), a nationally representative survey of people in South Korea. The annual KNHANES is administered by the Korea Disease Control and Prevention Agency (KDCA) using a multistaged systematic sampling method [25]. The present study included participants from the 2024 KNHANES survey cycle, during which serum Mg levels were measured. A flowchart depicting the sample selection procedure is presented in Figure 1. In total, 6033 adults were included in the 2024 KNHANES. We excluded pregnant women (n = 16), those with hepatitis B or C infection (n = 183), or those with liver cancer (n = 9), leaving 5825 individuals. After excluding individuals with missing data (n = 873), 4952 adults aged ≥18 years were analyzed. The 2024 KNHANES was performed with ethical approval from the Institutional Review Board of the KDCA (Approval No. 2022-11-16-R-0). Written informed consent was obtained from all participants.

### 2.2. Serum Mg Concentration

Blood samples were collected by venipuncture. A Cobas 8000 analyzer (Roche, Mannheim, Germany) was used to measure serum Mg levels (mg/dL). All measurements were within the clinically reportable range (0.30–9.60 mg/dL), and no values required imputation or replacement.

### 2.3. MASLD

Consistent with existing guidelines [1], MASLD was defined as the presence of hepatic steatosis with at least one of the five cardiometabolic abnormalities. Hepatic steatosis was identified using the Hepatic Steatosis Index (HSI) [26], a noninvasive index developed and validated in the Korean population. Multiple studies have demonstrated that HSI has reasonable diagnostic performance in detecting MASLD [27,28,29,30]. HSI was calculated using the following formula: 8 × (alanine aminotransferase-to-aspartate aminotransferase ratio) + body mass index (BMI), with an additional two points assigned for the presence of diabetes mellitus and two points for female sex. Consistent with previous studies [5,26], an HSI value of ≥ 36 was used to define hepatic steatosis.

The five cardiometabolic abnormalities considered in this study were as follows: (i) overweight or central obesity, defined as a BMI ≥23 kg/m^2^, corresponding to the World Health Organization Western Pacific Region criterion for overweight/obesity, or a waist circumference ≥90 cm in men or ≥85 cm in women; (ii) fasting plasma glucose ≥100 mg/dL, glycated hemoglobin (HbA1c) ≥ 5.7%, or the use of insulin or oral hypoglycemic agents; (iii) elevated blood pressure, defined as systolic/diastolic blood pressure ≥130/85 mmHg or the use of antihypertensive medications; (iv) hypertriglyceridemia, defined as triglyceride levels ≥150 mg/dL or the use of lipid-lowering medications; and (v) reduced high-density lipoprotein cholesterol (HDL-C), defined as HDL-C <40 mg/dL in men or <50 mg/dL in women, or the use of lipid-lowering medications.

### 2.4. Confounders

The following variables were included as potential confounders: sex, age, residential region (urban or rural), household income (lowest, low, high, or highest), educational attainment (middle school or below, high school, or college or above), marital status (married or unmarried), smoking status (yes or no), physical activity (yes or no), and alcohol consumption. Household income was categorized into quartiles based on the average monthly household income. Physical activity was classified according to the Korean version of the Global Physical Activity Questionnaire [31] as engaging in ≥150 min/wk of moderate-intensity physical activity, in accordance with the World Health Organization physical activity guidelines [32]. Alcohol use was categorized based on the Alcohol Use Disorders Identification Test-Consumption score [33]. Important confounders, including dietary Mg intake, supplement use, kidney function, diuretic or proton-pump inhibitor use, and other medications that may affect Mg homeostasis, were not addressed because the relevant information was unavailable.

### 2.5. Data Analysis

The association between serum Mg concentrations and MASLD was examined using logistic regression models. Serum Mg levels were analyzed as a continuous variable, with estimates expressed per 1–standard deviation (SD; 0.15 mg/dL) increment. Odds ratios (ORs) and 95% confidence intervals (CIs) were computed. Both the crude and adjusted models were fitted. Subsequently, the potential nonlinear association between serum Mg levels and MASLD was evaluated by restricted cubic spline functions with four knots [34]. Additionally, the relationship between serum Mg concentrations and each cardiometabolic risk was analyzed using the logistic regression models. It should be considered that the temporal sequence between serum Mg levels and MASLD could not be established in this study owing to the cross-sectional nature of the design.

We performed several sensitivity analyses. First, missing data were handled using multiple imputations with chained equations, and 20 imputed datasets were generated [35]. The imputation models incorporated the complex survey design of the KNHANES. Second, alternative hepatic steatosis indices, including the Framingham Steatosis Index [36] and the ZJU index [37], were used to define MASLD to assess the robustness of the findings (Table S1). Data analyses were conducted using the R (version 4.6.0). A complex survey design was accounted for using the survey package [38]. Logistic regression analyses were performed using the svyglm function. All regression models incorporated KNHANES sampling weights, strata, and clusters. Restricted cubic spline analyses were conducted using the rms package, and multiple imputation was performed using the mice package [35].

## 3. Results

Table 1 shows the sample characteristics. Overall, 56.6% of the participants were women, and the mean (SD) age was 53.16 (17.07) years. Among the participants, 25.7% (n = 1273) were classified as having MASLD, and 74.3% (n = 3679) as not having MASLD. The mean (SD) serum Mg concentration was 2.13 (0.15) mg/dL. The mean (SD) serum Mg concentration was 2.13 (0.15) mg/dL among participants without MASLD and 2.11 (0.15) mg/dL among those with MASLD.

Table 2 shows the relationship between serum Mg concentrations and MASLD. In the crude model, each 1–SD increase in serum Mg levels (0.15 mg/dL) was related to lower odds of MASLD (OR, 0.90; 95% CI, 0.85–0.96). After adjusting for potential confounders, the association remained largely unchanged (OR, 0.90; 95% CI, 0.84–0.96).

Table 3 shows the relationship between serum Mg concentrations and each metabolic risk factor. In the crude model, each 1–SD increase in serum Mg levels (0.15 mg/dL) was related to lower odds of increased glucose/HbA1c or DM medication use (crude OR, 0.93; 95% CI, 0.87–0.99). After adjusting for potential confounders, each 1–SD increase in serum Mg levels (0.15 mg/dL) was related to lower odds of overweight/obesity or high waist circumference (adjusted OR, 0.93; 95% CI, 0.87–0.99) and increased glucose/HbA1c or DM medication use (adjusted OR, 0.80; 95% CI, 0.74–0.86).

**Figure 2:**
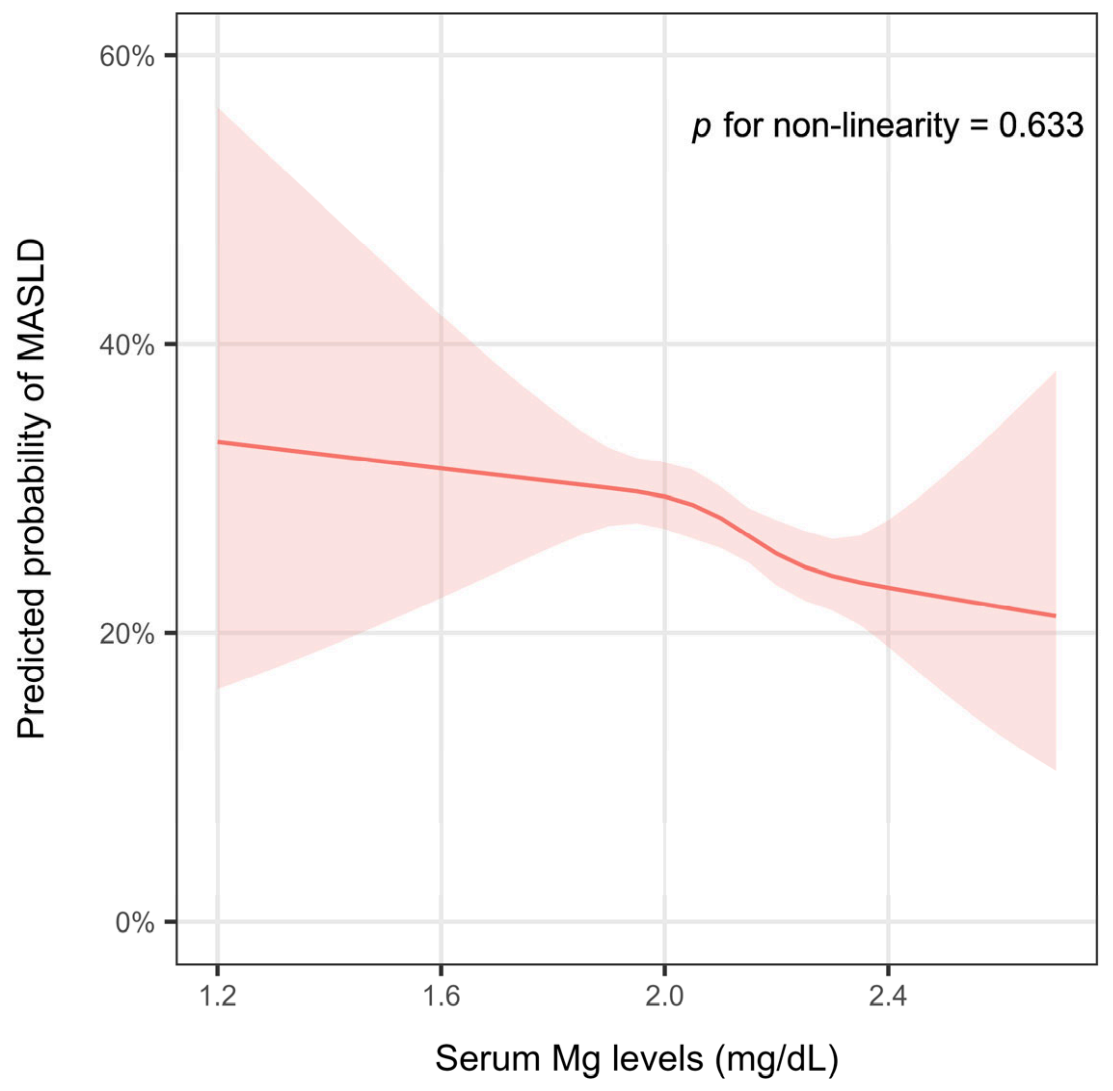
Nonlinear association between serum Mg concentrations and the predicted probability of MASLD using a restricted cubic spline function (MASLD, metabolic dysfunction-associated steatotic liver disease).

Figure 2 illustrates the dose–response relationship between serum Mg concentrations and the predicted probability of MASLD based on restricted cubic spline analysis. Serum Mg levels were inversely associated with the predicted probability of MASLD across the observed range. While the slope appeared to become steeper around serum Mg levels of 2.0–2.4 mg/dL, the test for nonlinearity was not significant (*p* for nonlinearity = 0.633).

Table S2 lists the results for the imputed datasets. The OR (95% CI) for the association between serum Mg levels and MASLD was 0.89 (0.84–0.95), which was consistent with the primary analysis. Table S3 presents the results of the sensitivity analyses based on alternative hepatic steatosis indices. The inverse association between serum Mg levels and MASLD remained consistent, with adjusted ORs (95% CIs) of 0.84 (0.79–0.90) based on the FSI and 0.88 (0.82–0.95) based on the ZJU index.

## 4. Discussion

This study aimed to examine the association of serum Mg levels with MASLD in adults. Utilizing a nationally representative sample, we revealed that serum Mg concentrations were inversely associated with MASLD. While the difference in mean serum Mg levels between the groups with and without MASLD was modest (2.13 vs. 2.11 mg/dL), even modest differences in serum Mg concentrations may be relevant at the population level. Therefore, the clinical significance of the observed difference should be interpreted cautiously. Although longitudinal studies using imaging-based diagnoses are warranted to establish temporality and confirm these findings, the present results provide important evidence regarding the association between Mg deficiency and MASLD.

These findings corroborate those of prior studies linking Mg deficiency or poor Mg intake to MASLD and NAFLD. Previous studies conducted among US adults have reported that dietary Mg intake is negatively associated with the prevalence of NAFLD [21,22]. Additionally, the Mg depletion score, a proxy measure of systemic Mg deficiency, is directly associated with the prevalence of MASLD among US adults [17]. Similarly, prior studies reported that low blood Mg levels are associated with a higher OR of NAFLD [23,39]. Building upon evidence from the existing literature, our study is among the first to report an inverse association between serum Mg concentrations and MASLD in a nationally representative Korean population.

The exact biological mechanisms underlying the connection between serum Mg levels and MASLD are not fully understood. However, previous studies have hypothesized that multiple pathways are involved in this connection [24,40,41]. Mg deficiency may disrupt lipid metabolism, resulting in elevated triglyceride and apolipoprotein B levels [42]. These alterations may increase hepatic lipid deposition, contributing to the development of hepatic steatosis and MASLD [40]. Among the metabolic abnormalities, the glucose/HbA1c outcome exhibited the strongest inverse association with serum Mg levels. Therefore, the association between Mg and MASLD may be driven mainly by glycemic dysfunction or insulin resistance. Indeed, previous studies have consistently indicated a link between Mg deficiency and insulin resistance [43,44]. Mg deficiency may contribute to metabolic dysfunction by impairing the secretion and activity of insulin. Dysregulation of intracellular Mg interferes with electrical activity of pancreatic β-cells [43]. Additionally, Mg deficiency can reduce tyrosine kinase activity and impair post-receptor insulin signaling, which may collectively facilitate insulin resistance, type 2 diabetes, and MASLD [44]. Additionally, Mg deficiency has been implicated in the development of oxidative stress and inflammation by increasing reactive oxygen species production and activating the nuclear factor-κB signaling pathway [45]. Consistent with these mechanisms, Mg deficiency is associated with chronic low-grade systemic inflammation, as reflected by increased C-reactive protein concentrations [46]. Persistent systemic inflammation promotes hepatic inflammatory responses, thereby accelerating the development and progression of MASLD [47].

The present study has some limitations. First, a causal relationship between serum Mg levels and MASLD could not be established because of the cross-sectional nature of the dataset. For instance, there is a possibility of reverse causation, in which MASLD can lead to hypomagnesemia via decreased absorption of nutrients and reduced synthesis of albumin [48]. Consequently, longitudinal studies are needed to analyze the temporal relationship between serum Mg levels and the subsequent onset of MASLD. Second, the assessment of hepatic steatosis was based on biomarkers rather than utilizing imaging modalities or biopsies. Although multiple studies have documented that HSI demonstrates reasonable diagnostic accuracy for MASLD [27,28,29,30], it remains susceptible to misclassification. Therefore, future studies should incorporate imaging-based assessments to improve the accuracy of MASLD assessment. Third, because of the lack of information, some potentially important confounding factors could not be controlled. In particular, data on dietary Mg intake and medications that may influence liver function or Mg homeostasis were not available, raising the possibility of residual confounding. Additionally, residual confounding by renal function, diet, and medication use should be considered. Fourth, the results from the study cannot be directly applicable to populations from other geographic regions. Fourth, because HSI and the cardiometabolic criteria for MASLD share common components, such as sex, diabetes, and BMI, this overlap may introduce potential circularity in the operational definition of MASLD. This limitation highlights the need for future studies using imaging modalities to address this issue.

## 5. Conclusions

This cross-sectional study reported that serum Mg concentrations were inversely associated with MASLD among a nationally representative sample of South Korean adults. However, whether serum Mg plays a preventive role in the development of MASLD remains to be established through longitudinal evidence. Therefore, future longitudinal studies incorporating imaging modalities are needed to establish temporality and confirm the relationship between serum Mg levels and MASLD observed in this study.

## Figures and Tables

**Table 1:** Characteristics of the study participants by MASLD status.

	Total	MASLD	
Variable		No	Yes
	N = 4952	N = 3679	N = 1273
Sex			
Male	2148 (43.38%)	1516 (41.21%)	632 (49.65%)
Female	2804 (56.62%)	2163 (58.79%)	641 (50.35%)
Age			
Mean (SD)	53.16 (17.07)	53.43 (17.44)	52.38 (15.95)
Region			
Urban	4033 (81.44%)	3019 (82.06%)	1014 (79.65%)
Rural	919 (18.56%)	660 (17.94%)	259 (20.35%)
Income level			
Lowest	851 (17.18%)	654 (17.78%)	197 (15.48%)
Low	1177 (23.77%)	847 (23.02%)	330 (25.92%)
High	1396 (28.19%)	1028 (27.94%)	368 (28.91%)
Highest	1528 (30.86%)	1150 (31.26%)	378 (29.69%)
Education level			
Middle school or below	1263 (25.50%)	929 (25.25%)	334 (26.24%)
High school	1639 (33.10%)	1217 (33.08%)	422 (33.15%)
College or above	2050 (41.40%)	1533 (41.67%)	517 (40.61%)
Marital status			
Married	3196 (64.54%)	2357 (64.07%)	839 (65.91%)
Unmarried	1756 (35.46%)	1322 (35.93%)	434 (34.09%)
Smoking status			
No	4266 (86.15%)	3189 (86.68%)	1077 (84.60%)
Yes	686 (13.85%)	490 (13.32%)	196 (15.40%)
Physical activity			
No	2747 (55.47%)	1951 (53.03%)	796 (62.53%)
Yes	2205 (44.53%)	1728 (46.97%)	477 (37.47%)
AUDIT-C			
Mean (SD)	3.42 (3.48)	3.37 (3.46)	3.54 (3.54)
Serum Mg levels (mg/dL)			
Mean (SD)	2.13 (0.15)	2.13 (0.15)	2.11 (0.15)

AUDIT-C, Alcohol Use Disorders Identification Test-Consumption; MASLD, metabolic dysfunction-associated steatotic liver disease; SD, standard deviation.

**Table 2:** Relationship between serum magnesium concentrations and MASLD.

	Crude Model	Adjusted Model
	OR (95% CI)	OR (95% CI)
Serum Mg levels		
Unit: 1–SD (0.15 mg/dL)	0.90 (0.85–0.96)	0.90 (0.84–0.96)
Sex		
Male		Reference
Female		0.53 (0.45–0.63)
Age		
Unit: 1–year		0.99 (0.90–1.00)
Region		
Urban		Reference
Rural		1.09 (0.89–1.33)
Income level		
Lowest		Reference
Low		1.21 (0.93–1.57)
High		1.11 (0.84–1.47)
Highest		0.99 (0.76–1.30)
Education level		
Middle school or below		Reference
High school		0.90 (0.73–1.11)
College or above		0.86 (0.68–1.08)
Marital status		
Married		Reference
Unmarried		0.81 (0.67–0.98)
Smoking status		
No		Reference
Yes		0.91 (0.74–1.12)
Physical activity		
No		Reference
Yes		0.63 (0.53–0.74)
AUDIT-C		
Unit: 1–point		0.99 (0.97–1.01)

AUDIT-C, Alcohol Use Disorders Identification Test-Consumption; MASLD, metabolic dysfunction-associated steatotic liver disease; OR, odds ratio; CI, confidence interval; SD, standard deviation.

**Table 3:** Relationship between serum magnesium concentrations and metabolic risks factors.

	Crude Model	Adjusted Model
	OR (95% CI)	OR (95% CI)
Serum Mg levels (Unit: 1–SD [0.15 mg/dL])		
**Outcome variables: Metabolic risk factors**		
1. overweight/obesity or high waist circumference	0.97 (0.92–1.04)	**0.93 (0.87–0.99)**
2. increased glucose/HbA1c or DM mediation use	**0.93 (0.87–0.99)**	**0.80 (0.74–0.86)**
3. elevated blood pressure	1.05 (0.98–1.12)	0.95 (0.88–1.02)
4. hypertriglyceridemia	1.01 (0.95–1.08)	0.94 (0.88–1.01)
5. reduced HDL-C	1.00 (0.94–1.07)	0.95 (0.88–1.02)

OR, odds ratio; CI, confidence interval; SD, standard deviation.

## Data Availability

Data are available at https://knhanes.kdca.go.kr/knhanes (accessed on 5 May 2026).

## Supplementary Materials

The following supporting information can be downloaded at https://www.mdpi.com/article/10.3390/metabo16080583/s1, Table S1: Alternative criteria for the classification of hepatic steatosis.; Table S2: Association between serum Mg levels and MASLD in logistic regression models. Sensitivity analysis using multiple imputation methods (n = 5825); Table S3: Association between serum Mg levels and MASLD in fully adjusted logistic regression models. Sensitivity analysis using alternative hepatic steatosis indices.
