# Supplementary material for: Association Between Serum Magnesium Levels and Metabolic Dysfunction-Associated Steatotic Liver Disease in a Nationally Representative Sample of Korean Adults, 2024
Source: Metabolites. 2026 Aug 18;16(8):583. doi: 10.3390/metabo16080583 (PMC13515035; doi:10.3390/metabo16080583)
Supplement: Supplementary file 1 [file metabolites-16-00583-s001.zip › metabolites-4476437-supplementary.pdf]

Table S1 Alternative criteria for the classification of hepatic steatosis.

Measures	Equation	Cutoff
FSI score[1]	−7.981 + 0.011 × age (years) − 0.146 × sex (female = 1, male = 0) + 0.173 × BMI (kg/m^2) + 0.007 × triglycerides (mg/dL) + 0.593 × hypertension (yes = 1, no = 0) + 0.789 × diabetes (yes = 1, no = 0) + 1.1 × ALT / AST ratio ≥ 1.33 × (yes = 1, no = 0).	> 23
ZJU index[2]	BMI (kg/m^2) + fasting glucose (mmol/L) + TG (mmol/L) + 3 × (ALT / AST) + 2 (if female)	> 38

[1] Long MT, Pedley A, Colantonio LD, Massaro JM, Hoffmann U, Muntner P, Fox CS. Development and Validation of the Framingham Steatosis Index to Identify Persons With Hepatic Steatosis. Clin Gastroenterol Hepatol. 2016 Aug;14(8):1172-1180.e2.

[2] Wang J, Xu C, Xun Y, Lu Z, Shi J, Yu C, Li Y. ZJU index: a novel model for predicting nonalcoholic fatty liver disease in a Chinese population. Sci Rep. 2015 Nov 16;5:16494.

Table S2 Association between serum Mg levels and MASLD in logistic regression models. Sensitivity analysis using multiple imputation methods (n = 5,825)

	**Crude model**	**Adjusted model**
	**OR (95% CI)**	**OR (95% CI)**
Serum Mg levels		
Unit: 1−SD	0.90 (0.84–0.95)	0.89 (0.84–0.95)

AUDIT-C, Alcohol Use Disorders Identification Test-Consumption; MASLD, metabolic dysfunction-associated steatotic liver disease; OR, odds ratio; CI, confidence interval; SD, standard deviation

Table S3 Association between serum Mg levels and MASLD in fully adjusted logistic regression models. Sensitivity analysis using alternative hepatic steatosis indices

	**FSI**	**ZJU index**
	**Adjusted OR (95% CI)**	**Adjusted OR (95% CI)**
Serum Mg levels		
Unit: 1−SD	0.84 (0.79–0.90)	0.88 (0.82–0.95)

AUDIT-C, Alcohol Use Disorders Identification Test-Consumption; MASLD, metabolic dysfunction-associated steatotic liver disease; OR, odds ratio; CI, confidence interval; SD, standard deviation
